# Millions of Bangladeshi Children Missed Their Scheduled Vaccination Amidst COVID-19 Pandemic

**DOI:** 10.3389/fpubh.2021.738623

**Published:** 2022-01-17

**Authors:** Sayed Manzoor Ahmed Hanifi, Nujhat Jahan, Nazia Sultana, Sharif-Al Hasan, Ashish Paul, Daniel D. Reidpath

**Affiliations:** Health System and Population Studies Division, International Centre for Diarrhoeal Disease Research, Bangladesh (icddr, b), Dhaka, Bangladesh

**Keywords:** COVID-19, expanded program on immunization (EPI), EPI outreach session (EOS), missing vaccine, Chakaria, Bangladesh

## Abstract

The Government of Bangladesh imposed a movement control order as a mass quarantine strategy to control the outbreak of coronavirus disease 2019 (COVID-19). Adherence to the home quarantine may put children at risk by missing routine vaccination. In this study, we investigated the impact of COVID-19 on child routine immunization in a rural area of Bangladesh and consider the broader implications. Data for this study comes from the Chakaria Health and Demographic Surveillance System (HDSS) of icddr,b with a population of 90,000 people residing in 16,000 households in 49 villages in a rural, coastal area of Southeast Bangladesh. We used an explanatory sequential mixed methods design which involved two phases between March 1, 2020, and May 31, 2020: first, we observed 258 outreach sessions of 86 EPI centers. We calculated the number of Expanded Program on Immunization (EPI) outreach sessions suspended and the number of children who missed their routine vaccination due to the COVID-19. We extrapolated the number of Bangladeshi children who missed their routine vaccination using Chakaria HDSS observations. Secondly, we conducted in-depth interviews to explain the quantitative results. The EPI outreach session (EOS) declined to 74.42% (95% CI 63.97–83.22), 10.45% (95% CI 5.00–18.94), and 3.45% (95% CI 1.00–9.75) from 2019 levels in March, April, and May 2020, respectively. By extrapolation, in Bangladesh, between March and May 2020, 3.2 million children missed their scheduled vaccination compared to 2019. Results from in-depth interviews showed that the unwillingness of villagers to hold EOS and the absenteeism of the vaccinators due to social distancing recommendations and lack of personal safety measures were the main reasons for the discontinuation of the EOS. Resuming EPI outreach sessions and introducing a special catch-up program is essential to prevent future outbreaks and deaths due to vaccine-preventable diseases in Bangladesh and the countries where children missed their routine vaccination due to COVID-19. This health system failure should be considered a factor in all future pandemic preparedness plans.

## Introduction

The declaration of the COVID-19 pandemic and the subsequent lock-down that occurred in many countries hindered the logistic supply chain for essential medicines and negatively affected the delivery of essential health services (1, 2). The situation may have been far worse for a country like Bangladesh to manage due to an unprepared or under-prepared health system response (2, 3). Bangladesh identified its first case on March 8th, 2020 (4). Nationwide general holiday was ordered after confirming the first 33 COVID-19 cases. This lockdown included complete shutdown of all government and private offices, educational institutions i.e., schools colleges, universities, local markets and shopping malls, and limiting all transportation systems including buses, trains, ships, and flights (5). Additionally, home and institutional quarantine was made compulsory by the government for the exposed individuals/overseas travelers (5). Until May 30th, the nationwide lockdown was extended several times. From June 1st, 2020 onwards, lockdown was partly relaxed and public could move around with ease (6). Lockdown policy was same for whole country. The spread of panic and misinformation; stigmatization of patients affected by the COVID-19 disease; the lack of personal protective equipment for health workers; inadequate numbers of health workers; travel limitations; and patient hesitation in leaving home, all negatively affected health-care seeking behavior (7, 8).

Due to the lockdown policy and limiting transportation, public was forced to maintain social distancing and avoid any crowdy places like the vaccination centers. Therefore, it is highly likely that the COVID-19 pandemic massively disrupted the EPI program with children missing important way-points in their routine vaccines (9). The empirical evidence, however, is patchy. Fortunately, health and demographic surveillance systems (HDSS) can provide the requisite evidence by virtue of their robust tracking of individual children in large community cohorts (10).

Using data from the Chakaria HDSS in a rural, coastal sub-district of Bangladesh, this study identifies the number of interrupted EPI Outreach sessions (EOS), the number of children who missed their routine vaccines, and underlying factors for obstructed EOS.

## Routine Vaccination in Bangladesh

In Bangladesh, the EPI project formally launched on 7th April 1979, as a pilot project but the vaccination centers were few and only located in urban healthcare facilities. As a consequence, the EPI coverage remained <2% until 1984 (11). The restructuring of service delivery to improve access followed findings perceiving that women might not reach immunization services if they had to travel long distances (12). So instead of converging vaccination in healthcare centers, the EPI immunization program has been mobilized to the selected house of villagers. Especially in remote areas where no community clinics or educational institutions available, the village chief's house or member's house or a small room in their housing area has been admitted as a voluntary contribution for the vaccination of the children of their community. The mobilization of the community to attend EPI assistance has been a significant factor contributing to the achievement of the program (12). Vaccination at rural wards is provided primarily by the Health Assistant (HA), an employee of the health wing of MOHFW, and is usually assisted by Family Welfare Assistant (FWA), an employee of family planning wing of MOHFW.

## Materials and Methods

### Settings and Population

The International Center for Diarrheal Diseases Research, Bangladesh (icddr,b) runs the Chakaria HDSS in Southeast Bangladesh. The HDSS covers a population of 90,000 individuals living in 16,000 households in 49 villages. All households are visited every 3 months to inquire into basic demographic events, including marriages, pregnancies, births, migrations, and deaths (13).

The EPI provides services in the HDSS area through 86 EPI outreach centers. Vaccines are provided according to the World Health Organization (WHO) recommended schedule: BCG (Bacillus Calmette–Guérin) at birth, Penta (diphtheria-tetanus-pertussis-hepatitis B-Haemophilus influenza type b) and OPV (oral polio vaccine), two doses of fIPV (fractional-dose inactivated polio vaccine) at 6 and 14 weeks of age. PCV (pneumococcal conjugate vaccine) in three doses at 6, 10, and 14 weeks of age, followed by measles vaccine (MV) at 9 and 15 months (Table 1). In the Chakaria HDSS area, of the children vaccinated, 95% are vaccinated through EPI outreach sessions (EOS) and the remaining 5% received their vaccinations from the local EPI head office located in Chakaria town. The coverage of fully immunized children by age 12–23 months was 84% in 2017 (14) which was similar to that of national estimate of 86% (15) It is noted, socio-economic, demographic and health indicators in Chakaria are similar to that of the national level (14, 15).

**Table 1:** Current vaccination (14 antigen) schedule in Bangladesh, 2020.

**Visit**	**Sequence of vaccination**
1st	BCG and OPV0 if baby visit within 2 weeks of age. Only BCG if s/he visits after 2 weeks of age to maintain the minimum recommended distance between two doses of OPV.
2nd	Penta1, PCV1, OPV1, and fIPV1 at 6 weeks of age.
3rd	Penta2, PCV2, and OPV2 at 10 weeks of age.
4th	Penta 3, PCV3, fIPV2, and OPV3 at 14 weeks of age.
5th	First dose of MR at 9 months of age.
6th	2nd dose MR at age of 15–18 months.

*Penta and OPV are administered with a minimum gap of 4 weeks*.

In the HDSS, data on the vaccination of individual children below 3 years of age is collected during trimonthly household visits. The monthly vaccination schedule of the 86 EPI outreach centers is collected from the local EPI office once a year. Data are also collected on whether the EPI sessions were conducted at the scheduled date and if the session did not occur, the reasons.

### Statistical Methods and Analysis

Here we used an explanatory sequential design where the first step started with collection and analysis of quantitative data and in the second step to explain quantitative findings, we have used the qualitative methods as the follow up (16). This combination of qualitative and quantitative method is extensively used in health and social science related studies (17, 18).

**Figure 1:**
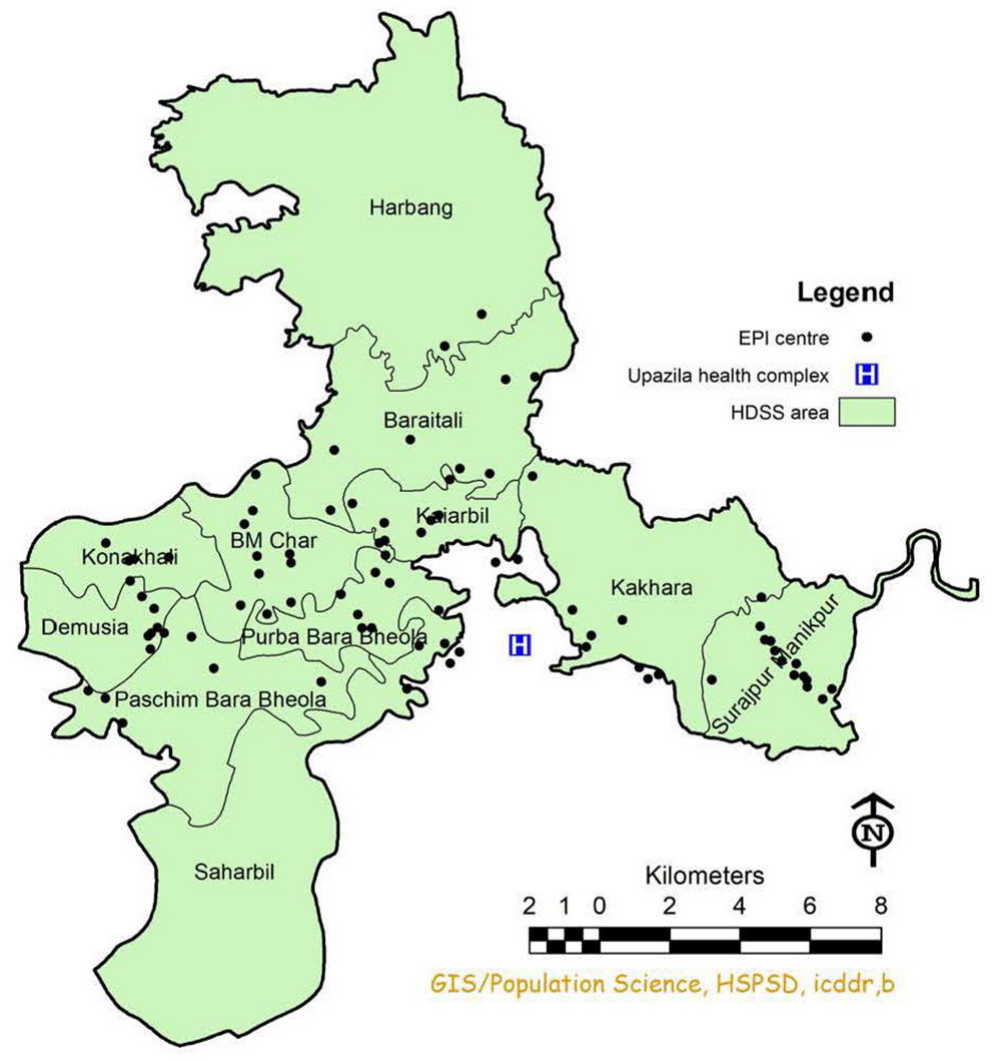
Map showing EPI centers in Chakaria HDSS area.

Our mixed method study had two phases, quantitative and qualitative between March 1, 2020, and May 31, 2020 (COVID-19 pandemic movement control period). Firstly, we observed 258 outreach sessions in 86 EPI centers and counted the number of suspended EOS (Figure 1). The percentage of sessions carried out during the pandemic period was compared with the sessions held during the equivalent period in 2019 (March-May). Secondly, of the study was done to understand the underlying factors for any disrupted sessions from both a provider and a community perspective. In this phase, in-depth interviews were conducted with vaccinators (*n* = 6) and villagers whose houses were used for holding EOS (*n* = 16).

From the HDSS data trend, we have observed that the number of children who are vaccinated was approximately the same in 2018 and 2019. We calculated the average number of children vaccinated (14 antigens, Table 1) during the period March to May between 2018 and 2019 through 86 EPI centers for 2,186 newborns of Chakaria HDSS area (Table 2). Then, we estimated the number of children who missed their vaccinations from March to May 2020 by the proportion of sessions suspended. Finally, we extrapolated the number of children who missed their scheduled vaccine in the pandemic period based on the 324,000 immunization sessions in Bangladesh (19) (Table 3).

**Table 2:** Number children vaccinated during 2018 and 2019.

**Month**	**2018**	**2019**	**Average number of children vaccinated between 2018 and 2019**
March	1,145	1,060	1,103
April	1,086	1,054	1,070
May	1,145	933	1,039
Number of newborns	2,116	2,256	2,186

**Table 3:** Number of children missed EPI session between March and May, 2020.

**Site**	Number of newborns^*^	Number of outreach EPI center^**^	**Average number of children vaccinated through EPI session between 2018 and 2019**			**Number of children missed vaccination session in 2020**			
			**March**	**April**	**May**	**March**	**April**	**May**	**Total number of children (95% CI)**
Chakaria HDSS	2,256	86	1,103	1,070	1,039	282	958	1,003	2,243 (2,191–2,294)
Bangladesh	3,087,626	108,000	1,509,597	1,464,433	1,422,005	386,155	1,311,399	1,372,946	3,168,824 (3,166,907–3,170,740)

* *United Nations, Department of Economic and Social Affairs, Population Division (20)*.

** *Khan and Richard (19)*.

## Results

### Observation of EOS

Twenty-seven vaccinators carried monthly EPI sessions. Out of 86 centers, 64 were in villagers' house, 14 in community clinic, 1 in health facility and 7 in educational institutions. Children visited EPI centers 5–6 times to receive the full complement of 14 routine antigens. Whether it was five or six times depended on the sequence of the BCG and Penta vaccines. The average number of children vaccinated in a session was 12 (95% CI 11–13).

#### Missing EPI Sessions

**Figure 2:**
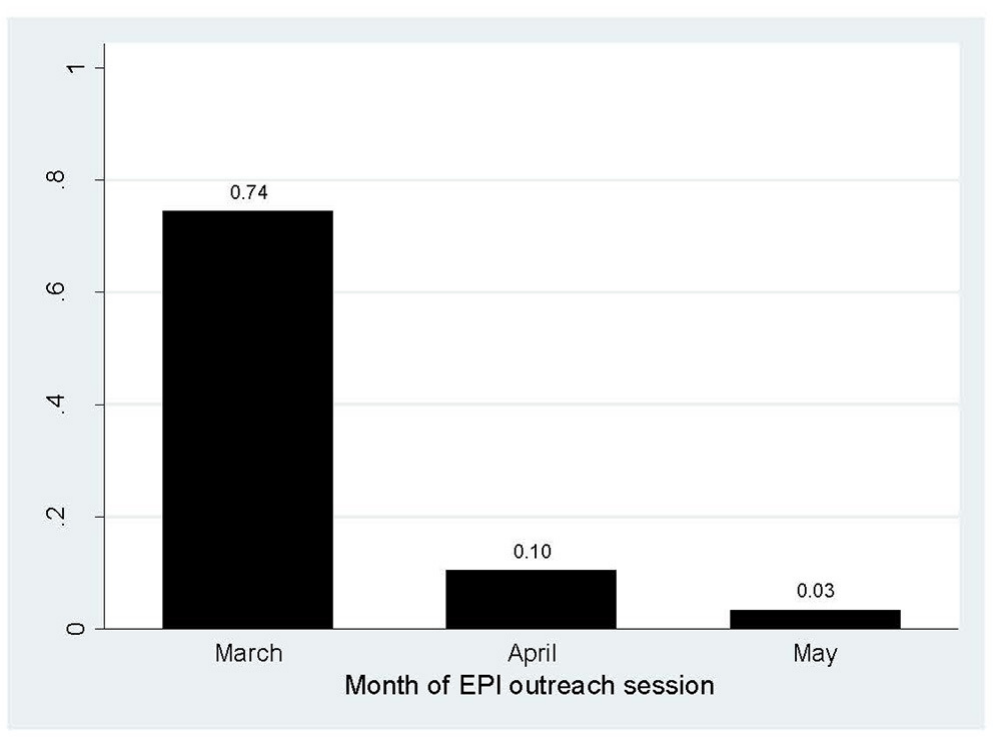
Percentage of EOS conducted by month, 2020.

In 2019, 99% (CI 98.37–99.76) of 86 planned monthly EOS were carried out in the Chakaria HDSS area. In 2020, the EPI outreach session declined dramatically. The figure was 74% (95% CI 64–83), 10 % (95% CI 5–19), and 3% (95% CI 1–10) in March, April, and May, respectively (Figure 2).

### Children Missing Vaccinations

Table 2 presents the number of children vaccinated between 2018 and 2019. On average, 1,071 children were vaccinated per month; vaccination varied for month and year. Compared to 2018–2019, 2,243 (95% CI 2191–2294) children missed vaccination session between March 1, 2020 and May 31, 2020 in scheduled 258 sessions of 86 EPI centers. Extrapolated up, in Bangladesh, 3,168,824 (95% CI 3,166,907–3,170,740) children missed their routine vaccination sessions (Table 3).

### Reasons for Not Conducting EOS

Some of the owners of the houses where EPI centers have been set up complied stringently with the government lock-down policy; some said vaccinations should continue, with appropriate safety measures, and some said they have no problem continuing, but the vaccinators themselves attended fewer sessions.

One of the owners of a center reflected on the possible problems arising from the lock-down policy.

*“Now everything is closed due to the lock-down. The Government has forbidden us to go to another house. During vaccination, problems might arise when different people come.”*

In some vaccination centers, homeowners expressed concerns that due to the vaccination program, there would be crowds that could possibly spread Coronavirus. They warned that the vaccinators had to vaccinate children a safe distance from their houses and would no longer allow the vaccinators to sit in their houses.

In contrast, some of the respondents said there was no harm in holding the EPI in their house.

*“Vaccination staff sit in a small room in front of the house. So, we have no objection to vaccination.”*

Respondents who had separate rooms had no objection to the vaccination program as the family can keep a safe distance from the crowd. They have to be a little more careful, such as when there are parents with children and they have to wear a mask and have to maintain distance, and the vaccinator has to sit outside.

Additionally, the other respondent said:

*“There is no problem if vaccinator comes. Moreover, the vaccination staffs themselves are not coming to vaccinate for 2 months.”*

If the children of a village are not appropriately vaccinated, they may become sick, and the entire community will face the consequences. The house owners do not believe in viruses, but they understand the importance of vaccination, so they have ensured that there will be no problem in their houses. However, they have pointed out that due to lock-down, the vaccinators are not coming for a period of 2 months.

The reason for not conducting the EPI session, however, is mainly due to the vaccinator and not the house owner. As one of the respondents reported that

*“The vaccinator had called me and said, worldwide Coronavirus is spreading, so I am not going to vaccinate now. If mothers bring their children, please tell them I will vaccinate later.”*

Due to lock-down, the vaccinator might face difficulties with transportation, and there were no proper guidelines developed for vaccinators about how to continue the vaccination program. Also, some of the vaccinators did face difficulties from the house owners and members of the villages. As a result, they became distressed and postponed their sessions.

One Health Assistant said the lack of PPE and the house owners' fear of getting affected with COVID 19 was the reason they stopped attending the EOS.

*“I did not go to the vaccination center since March 17. Because our office did not give PPE; it is not safe to work without PPE. The government gave us just two pairs of gloves and a mask. Nevertheless, I went to the house last week, but they did not let me do the vaccination because they think that if more people gathered, the homeowner would be at risk of getting affected with Corona.”*

It is essential to provide proper PPE to healthcare workers who were in direct contact with the community because it is they who could become the super spreaders. Nonetheless, due to mismanagement, the health assistants did not receive proper PPE. They still tried to resume the vaccination process. However, the homeowners were afraid that if people gathered in their house, they might become get COVID-19.

A health vaccinator reported the objection of house owners and villagers due to imposed lock-down by the government.

*“After about 1 month, I went to the vaccination center on April 26, 2020. Villagers told me that the government instructed to keep the distance during lockdown. If you come, won't we face any problems? I tried to vaccinate the kids from a certain distance, but the public did not keep the distance. That day I could vaccinate only five children”*

Some homeowners expressed strong reluctance to hosting the vaccinators, as the government had asked everyone to be aware of the COVID-19 situation. Coronavirus is spreading all over the world, and everything is closed, so the homeowners think that the vaccination center should also be closed. Despite facing these problems, the vaccinator still tried to vaccinate children while maintaining appropriate social distancing, but people gathered and did not maintain the distance.

Another Health Assistant expressed his experience differently.

*“We faced some problems in launching the sessions, as the homeowners and the locals created obstacles. They said the government has locked down everything; now you are coming in contact with people again. The EPI center was closed for 1 month then we continued seven sessions. Later, on April 30, as two staff from the Chakaria Thana Health Complex got infected by COVID-19. Although people who came in contact with those two corona patients were tested negative, we stopped all the activities of the field until the second of May 2020. The EPI sessions will start again if Sir provides direction.”*

## Discussion

### Main Findings

From March to May 2020, 71% of the EOS were suspended; 2,116 children missed their routine vaccines in 86 EPI centers in the study area. The unwillingness of villagers to hold EPI outreach sessions and the absenteeism of the vaccinators were the main reasons for the discontinuation of the EOS. Due to social distancing recommendations and not receiving guideline to continue the session increased the absenteeism of vaccinators. They also faced lack of personal safety measures for themselves which also obstructed conducting EOS sessions.

#### Interpretation and Policy Implications

Prior to the pandemic, Bangladesh had been providing immunization services in the rural areas through around 108,000 immunization sessions held every month (19). Extrapolating from our data shows that about 3.2 million children missed out on vaccinations for BCG, Penta, OPV, IPV, PCV, and MV in 3 months of the pandemic lock-down in 2020.

The reasons for disrupting immunization sessions are related to the interplay of multiple factors. First of all, health care services have stretched and directed to other priorities due to COVID19. This is consistent with the findings from WHO report (21) and studies including in Bangladesh (22) and LMICs (23). The second reason is that social distancing recommendation, vaccinators were not attending in EPI session as they felt unsafe without PPE. Social distancing and unavailability of vaccinators are found as reasons for immunization disruptions in Nepal, Senegal and Liberia (24), and other LMICs (23). Thirdly, the majority of the EPI session held in a house owned by the villagers and vaccinators were unaware whether the owner of the household agrees to vaccinate children in their house in the pandemic. Findings from a rapid assessment on impact of COVID-19 essential health services in Bangladesh suggest that in around one third cases, vaccination centers were not placed at the right points (24). Finally, the EPI program personnel thought that the pandemic period of coronavirus disease would be temporary and the service providers did not pay attention to this and they had not taken any plan to continue vaccination in COVID-19 pandemic situation.

Moreover, the service providers were not ready to continue vaccination at the community level to protect vaccinators as well as community people both house-owner of the EPI center and mothers who bring their children in EPI center, and the children. Therefore, decision-makers around strategies needs to prepare an ideal policy to sustain routine immunization services provided through EPI centers with the engaging local community in a rural area like Bangladesh in the pandemic period like COVID-19.

In addition, large-scale budget should support its structure to work with the World Bank and other multilateral advancement banks to defend public expenditure on EPI vaccines delivery programs (25). The budget should include the purchase of adequate personal protective equipment for health workers, vaccinators, and all community-level field workers. EPI vaccination programs are an essential public health expenditure that should ideally be funded extensively by a government's spending even in low-income countries and an essential part of pandemic preparedness as well (25).

During the Ebola virus outbreak, vaccine-preventable diseases such as measles and pertussis increased in countries where the primary pediatric healthcare services were halted or reduced (26). The infants and young children were observed as more vulnerable age groups for infectious diseases during the Spanish Flue and Black Death (27, 28).

Measles vaccines have recently been shown to strengthen the immune system to fight off pathogens other than measles, through a *bystander immunity*, which also appears to intensify the immune response to the novel Coronavirus (29). Moreover, the health benefits of childhood vaccination appear to outweigh the risk of contracting and dying from COVID-19 during immunization clinic appointments (25).

Bangladesh is one of the countries where immunization have delayed due to pandemic and resurgence of Diphtheria and cholera has been reported in several places (30, 31). Therefore, a systematic action is needed as a catch-up activity to monitor the immunization sessions. This process may include identifying children who missed immunization and providing proper PPE and training to the health workers. It is also essential to build trust by maintaining timely childhood vaccinations while protecting both vaccinators and the community people. This can be done within the communities by involving community leaders, public and private organizations (including NGOs), creating awareness with appropriate knowledge and safety measures, and engaging in community mobilization and sensitization activities.

### Strength and Weakness

This study warns about the suspension of EOS in rural areas due to the current COVID-19 pandemic situation using data collected through the HDSS platform. To measure the impact of COVID-19 on childhood routine immunization, we used mixed methods research design (32). This included observations of EOS as well as in-depth interviews, of providers and beneficiaries, to understand the complexity and challenges involved in providing EPI services during the pandemic. The study, carried out in a rural area, may not represent the scenario of Bangladesh. However, the impact of the lock-down policy on the interruption of the EPI outreach sessions is likely to be similar (at least in direction) across the country.

## Data Availability Statement

The raw data supporting the conclusions of this article will be made available by the authors, without undue reservation.

## Ethics Statement

The studies involving human participants were reviewed and approved by Ethical Review Committee of International Center of Diarrhoeal Disease Research, Bangladesh. Written informed consent for participation was not required for this study in accordance with the national legislation and the institutional requirements.

## Author Contributions

SH and DR conceived and designed the study. S-AH and AP prepared the data file. SH analyzed the data and wrote the first draft of the manuscript. NJ and NS assisted in the literature review. All authors contributed to the final version of the manuscript.

## Funding

This research study was funded by core donors who provide unrestricted support to icddr,b for its operations and research. Current donors providing unrestricted support include the Governments of Bangladesh, Canada, Sweden, and the UK.

## Conflict of Interest

The authors declare that the research was conducted in the absence of any commercial or financial relationships that could be construed as a potential conflict of interest.

## Publisher's Note

All claims expressed in this article are solely those of the authors and do not necessarily represent those of their affiliated organizations, or those of the publisher, the editors and the reviewers. Any product that may be evaluated in this article, or claim that may be made by its manufacturer, is not guaranteed or endorsed by the publisher.
