# Supplementary material for: Millions of Bangladeshi Children Missed Their Scheduled Vaccination Amidst COVID-19 Pandemic
Source: Front Public Health. 2022 Jan 17;9:738623. doi: 10.3389/fpubh.2021.738623 (PMC8801521; doi:10.3389/fpubh.2021.738623)
Supplement: Supplementary file 1 [file Data_Sheet_1.PDF]

Supplementary Table 1. The extrapolation process to calculate the number of children missed vaccination session in 2020

Average of number of children vaccinated through EPI session between 2018- 2019. Bangladesh. ((Average of number of children vaccinated through EPI session between 2018-2019 Chakaria HDSS/Number of newborn Chakaria HDSS) *(Number of newborns in Bangladesh))	March	April	May	Total Number of Children
	1,509,597	1,464,433	1,422,005	
Percentage of Children Vaccinated 2020 (Figure 2.)	74.42	10.45	3.45	
Percentage of Children Not Vaccinated in 2020	25.58	89.55	96.55	
Number of children missed vaccination session in 2020 Chakaria HDSS (Average of number of children vaccinated through EPI session 2018-2019 * % of children Not Vaccinated 2020)	282	958	1,003	2243
Number of children missed vaccination session in 2020 Bangladesh. (Average of number of children vaccinated through EPI session 2018-2019 * % of children Not Vaccinated 2020)	386,155	1,311,400	1,372,946	3070500
